# Wide Angle of Incidence-Insensitive Polarization-Independent THz Metamaterial Absorber for Both TE and TM Mode Based on Plasmon Hybridizations

**DOI:** 10.3390/ma11050671

**Published:** 2018-04-25

**Authors:** Xiu Tao Huang, Cong Hui Lu, Can Can Rong, Sheng Ming Wang, Ming Hai Liu

**Affiliations:** 1 State Key Laboratory of Advanced Electromagnetic Engineering and Technology, Huazhong University of Science and Technology, Wuhan 430074, China; xthuang@hust.edu.cn (X.T.H.); ccrong@hust.edu.cn (C.C.R.); smwang@hust.edu.cn (S.M.W.); 2 School of Physics, Huazhong University of Science and Technology, Wuhan 430074, China; conghuilu@hust.edu.cn

**Keywords:** metamaterial absorber, wide angles of incidence, plasmonics, single band, broadband, sixteen-circular-sector (SCR) resonator

## Abstract

An ultra-wide-angle THz metamaterial absorber (MA) utilizing sixteen-circular-sector (SCR) resonator for both transverse electric (TE) and transverse magnetic (TM) mode is designed and investigated numerically. At normal incidence, the absorptivity of the proposed MA is higher than 93.7% at 9.05 THz for different polarization angles, due to the rotational symmetry structure of the unit cell. Under oblique incidence, the absorptivity can still exceed 90%, even when the incident angle is up to 70° for both TE and TM mode. Especially, the frequency variation in TE mode is less than 0.25% for different incident angles from 0° to 70°. The electric field (*E_z_*) distributions are used to explain the absorption mechanism. Numerical simulation results show that the high absorption with wide-angle independence stems from fundamental dipole resonance and gap surface plasmons. The broadband deep-infrared MA is also obtained by stacking three metal-dielectric layers. The designed MA has great potential in bolometric pixel elements, biomedical sensors, THz imaging, and solar cells.

## 1. Introduction

Electromagnetic (EM) metamaterials, which are composed of artificial sub-wavelength materials (so called meta-atom or meta-molecules) and present exotic properties not found in nature, have been paid widespread attention in the frequency range from visible to microwave band [1,2,3,4]. By tailoring the effective complex permittivity (ε = ε′ + iε″) and permeability (μ = μ′ + iμ″), EM metamaterials possess unique functionalities and great potential in super lenses [5,6,7], invisible cloaks [8,9,10], polarization convertors [11,12,13], solar cells [14,15,16], and absorbers [17,18,19].

In particular, metamaterial absorber (MA) has been extensively investigated, as was demonstrated by Landy et al. in 2008 [17]. Then, Tao et al. presented THz MA in the terahertz (0.1–10 THz) region, which was composed of a top split-ring resonator and a bottom cut-wire metal separated by a dielectric layer [18]. Due to the bottom cut-wire metal, it is difficult to design high absorption MA. To overcome this issue, a continuous metallic ground film instead of the cut-wire metal is introduced into perfect MA in the later research [20,21,22]. Since then, all kinds of types of THz MA, such as single band [22], dual band [23], three band [24], multiband [25,26,27], broadband [28,29], polarization controlled [30], polarization-insensitive [31,32], and incident angle-insensitive [33,34,35] operations, have been achieved and experimentally demonstrated. For example, to increase the number and width of absorption band, there are usually three main ways, including non-planar stacking layers [35], combination of different metallic patches in the same plane [36], and a simple structure combined with different resonant modes [26]. Polarization property of MA is dependent on the geometrical structure of unit cell. MA is polarization-insensitive only if the unit cell is rotational symmetrical structure. In addition, the absorptivity of THz MA is usually related to incident angle [35,36,37,38,39]. However, it is more difficult to create the high absorption MA with angle-insensitive characteristic. Nowadays, to obtain the wide-angle absorption, different resonance structures of the unit cell have been designed. Tao et al. numerically and experimentally investigated the wide-angle stability of the single band MA based on the split ring resonators [22]. Liu et al. presented wide-angle THz MA by stacking metallic bars, and the absorptivity exceeds 90% below 40° angle of incidence [28]. Zhu et al. proposed the THz MA based on square patches, and the absorption intensity decreases with the increase of the incident angle [29]. He et al. numerically demonstrated the wide-angle properties of THz MA using saw-shaped annular patches resonator below 50° [33]. Zhang et al. reported a cross-shaped graphene MA, which can achieve the wide-angle absorption for TM mode [37]. However, the previous THz MA showed angle-insensitive absorption with greater than 90%, only in the case of TM mode. Moreover, their absorptivity and absorption bandwidth decreased with the increase of incident angle in TE mode. Therefore, an angle-insensitive MA for TE configuration is very desirable in the terahertz range.

In this work, we design an ultra-wide-angle-independent, polarization-insensitive MA in the terahertz region. The proposed single-band MA consists of sixteen-circular-sector (SCR) metallic-dielectric resonators on a continuous metallic film. Numerical simulations demonstrate that more than 90% absorption is still obtained under oblique incidence up to 70° for TE and TM mode. Moreover, the frequency variation under TE mode is smaller than that under TM mode. Based on the aforementioned study, we present broadband MA in the deep infrared range. To our best knowledge, the designed single band MA with angle-insensitive absorption has great practical applications, such as in bolometric pixel elements and biomedical sensors.

In Section 2, the design and simulation process of the MA is described. The simulated results concerning the influence of some geometrical parameters on the absorptivity are presented in Section 3. The lossy characteristics, the different parameter values, wide incident and polarization angles, and the electric field and current distributions, are discussed in detail. Finally, Section 4 summarizes the conclusion.

## 2. Design and Simulation

**Figure 1:**
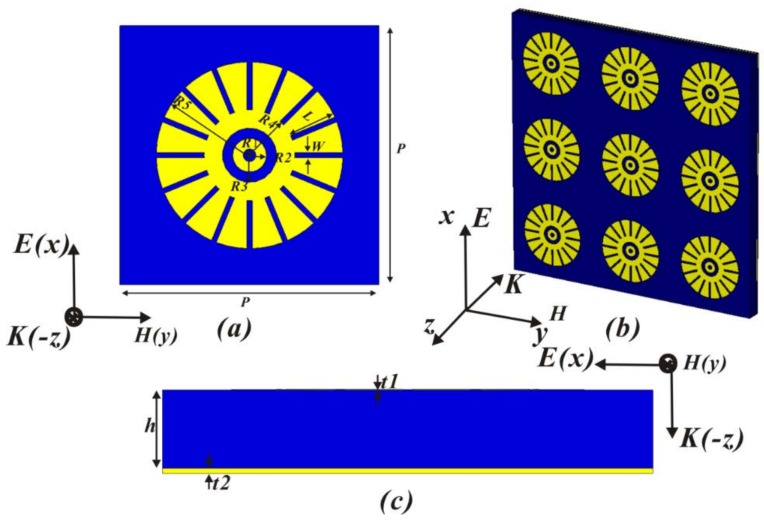
(**a**) Top view of unit cell of THz metamaterial absorber (MA) with sixteen-circular-sector resonator, (**b**) 3D view of THz MA, (**c**) cross section of unit cell.

The structure of the proposed single-band THz MA is shown in Figure 1. The designed MA is composed of periodic sixteen-circular-section (SCR) patterns and a continuous metallic ground film separated by dielectric spacer. For ensuring good conductivity, two metallic layers (SCR patterns and ground film) are made of gold with conductivity of 4.56 × 10^7^ S/m. A lossy polymer is set as the dielectric layer with complex permittivity (ε = 3.5 + 0.2i) in the simulation [40]. Figure 1a,c show top view and cross section of unit cell with the final dimensions (*R1* = 0.45 μm, *R2* = 0.65 μm, *R3* = 1.05 μm, *R4* = 1.75 μm, *R5* = 3.6 μm, *w* = 0.2 μm, *P* = 10 μm, *h* = 1.6 μm, *t1* = 0.01 μm, and *t2* = 0.1 μm). The thickness of the top metallic layer is thinner. Figure 1c shows the periodic arrangement of 3 × 3 unit cells of THz MA for clarity. The above geometric parameters are optimized by the full-wave simulations using computer simulation technology (CST) Microwave Studio, where a unit cell is simulated by the incident THz plane waves radiation along the *−z* direction, with electric field (E) along the *x* direction and magnetic field along the *y* direction. The boundary conditions are unit cell in the *x* and *y* directions, and Floquet ports are used in the *z* direction. The absorptivity (A(*w*)) is characterized by using A(*w*) = 1 − T(*w*) − R(*w*). T(*w*) and R(*w*) are transmission and reflection, respectively. T(*w*) in the simulation is zero, because the thickness of a continuous metallic film is greater than its skin depth. To minimize the reflection R(*w*), the effective impedance (Z) of THz MA matches that (Z_0_) of free space (Z = Z_0_) by tuning geometrical parameters of the system to manipulate the effective ε and μ. The resonant property of the MA can be analyzed by an equivalent inductance and capacitance circuit model, the resonance frequency based on an *LC* circuit model is usually used as follows [40,41]:

(1) $$f_{m}=\frac{1}{2\pi \sqrt{LC/2}}∼\frac{1}{l}$$

where *C* is the effective capacitance, *L* is the effective inductance, and *l* is the effective length in sixteen-circular-section resonator.

## 3. Results and Discussion

**Figure 2:**
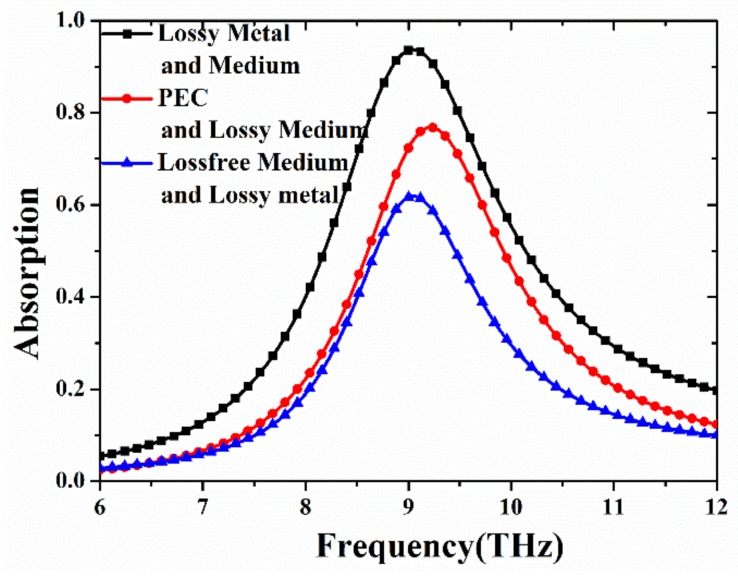
Dependence of absorption spectra of the proposed THz MA on different material properties of PEC (perfect electrical conductivity) and loss-free medium.

To analyze the loss distributions of each part of MA, the absorptivity of the proposed MA in three loss properties is displayed in Figure 2. Clearly, a single resonance peak is obtained in the THz frequency range. In the case of lossy metal and material, the absorptivity achieves 93.7% at the resonant frequency of 9.05 THz. The thickness of the MA is only *λ*/21, where *λ* is the resonance wavelength. When the PEC (perfect electrical conductivity) and lossy medium is used in the system, its absorptivity reaches 76.8% at 9.225 THz. As for case of loss-free medium and lossy metal, the absorptivity of 61.88% at 9.05 THz is obtained. Moreover, it can be seen that the resonance frequency is shifted when PEC is introduced into the MA. However, the position of the resonance peak is fixed when the loss properties of the medium change, which is consistent with previous study [27,42]. Therefore, the above results indicate that the high absorption of the proposed MA depends on both the dielectric loss and ohmic loss. Also, the dielectric loss caused by the lossy polymer is slightly greater than ohmic loss within the metal. Thus, the results disagree with previous research [27,42], where the intensity of dielectric losses is at least an order of magnitude higher than that of ohmic losses. In references [27,42], about 80% of the energy is consumed by the dielectric substrate, and only 20% of the energy is dissipated in the metal layers. It is noted that the absorption curve of PEC (red line) is consistent with that of loss-free medium (blue line) in the low frequency range from 6 THz to 7 THz. The reason is that dielectric and ohmic losses have little influence on the absorptivity, due to the small off-resonance absorption. Compared with the original MA, the absorptivity under the conditions of PEC (red line) and loss-free medium (blue line) is much lower due to the reduced losses. Therefore, there appears to be no difference in the low frequency range from 6 THz to 7 THz.

**Figure 3:**
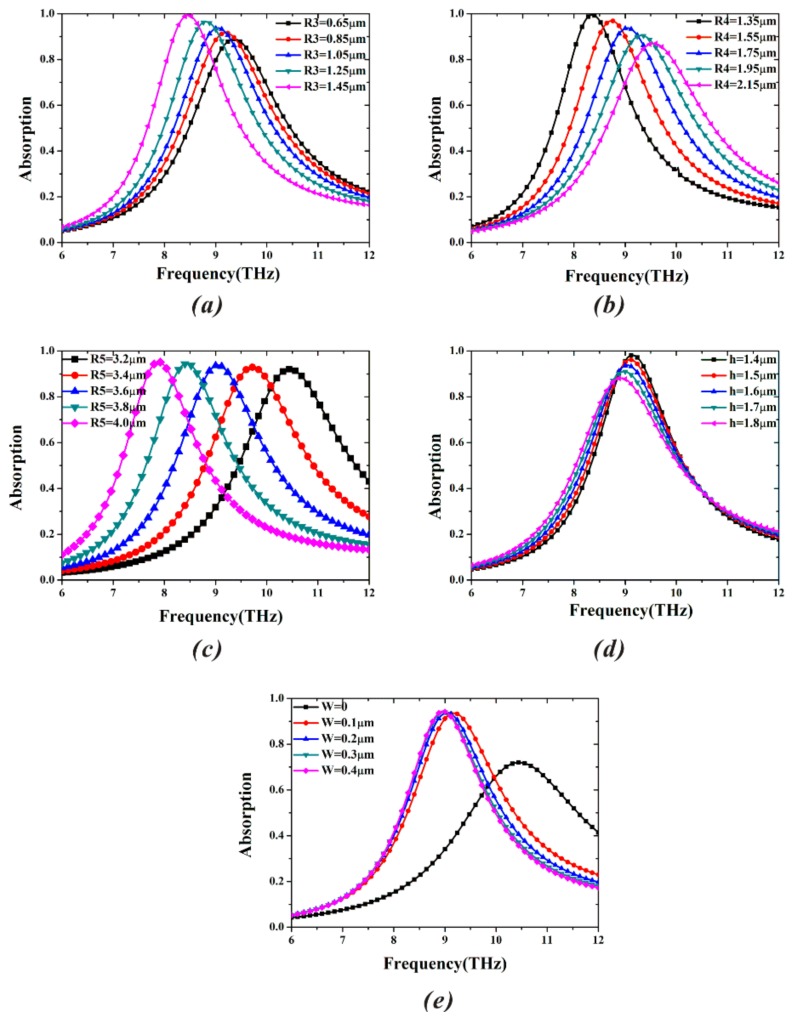
Simulated absorption spectra of the proposed MA under different geometric parameters: (**a**) the inner radius (*R3*) of sixteen circular sector at the center (*R1* = 0.45 μm, *R4* = 1.75 μm, *R5* = 3.6 μm, *h* = 1.6 μm, and *W* = 0.2 μm), (**b**) the inner radius (*R4*) of sixteen circular sector (*R1* = 0.45 μm, *R3* = 1.05 μm, *R5* = 3.6 μm, *h* = 1.6 μm, and *W* = 0.2 μm), (**c**) the outer radius (*R5*) of sixteen circular sector (*R1* = 0.45 μm, *R3* = 1.05 μm, *R4* = 1.75 μm, *h* = 1.6 μm, and *W* = 0.2 μm), (**d**) the thickness (*h*) of dielectric layer (*R1* = 0.45 μm, *R3* = 1.05 μm, *R4* = 1.75 μm, *R5* = 3.6 μm, and *W* = 0.2 μm), and (**e**) the width (*w*) of gap in the sixteen circular sector (*R1* = 0.45 μm, *R3* = 1.05 μm, *R4* = 1.75 μm, *R5* = 3.6 μm, and *h* = 1.6 μm).

To obtain the high absorption, just studying the impacts of the dielectric and ohmic losses on absorptivity is not enough, because the change of the geometric parameters of MA can directly influence the near-field coupling, and consequently, tune the resonance characteristics. At normal incidence, the effects of different geometric parameters (*R3*, *R3*, *R5*, *h*, and *w*) on the absorption spectra of the proposed MA are depicted in Figure 3. In Figure 3a, when *R3* increases from 0.65 μm to 1.45 μm, the absorptivity gradually increases and the resonance frequency experiences redshift. The increase of radius (*R3*) makes the effective length (*l* in Equation (1)) bigger, leading to the reduction of resonance frequency. As shown in Figure 3b, conversely, the absorption ratio (always beyond 85%) gradually decreases, while the resonance frequency has an obvious blueshift as *R4* increases. The reason is that the increase of radius (*R4*) can make the value of geometric parameter (*L*) in Figure 1a smaller, resulting in reduction of the effective capacitance (*C*), and further leading to the increase of the resonance frequency. When the outermost radius (*R5*) increases from 3.2 μm to 4.0 μm in steps of 0.2 μm in Figure 3c, the resonance peak with the same absorption coefficient shifts to lower frequency (redshift), and the frequency variation also remains the same. Obviously, resonance frequency is linearly associated with outermost radius (*R5*). The absorptivity exceeds 90% in the entire range. The absorption bandwidth, which is defined as a full width at half maximum (FWHM), is 2.05 THz at the resonant frequency of 9.05 THz. Therefore, the adjustable *R5* in the proposed MA provides the potential in the biosensor. In Figure 3d, the thickness of the polymer in MA has a slight influence on the properties of the absorption peak. It is found that the absorptivity is always greater than 90% when the thickness (*h*) changes from 1.4 μm to 1.7 μm. Furthermore, the increase of the dielectric thickness makes the absorption frequency redshift, and results in the decrease of the absorptivity. In Figure 3e, when the value of gap (*w*) varies from 0 μm to 0.4 μm, the circular ring resonator becomes the sixteen-circular-section one, leading to increased capacitance. Thus, the resonance frequency abruptly decreases with the increase of the gap (*w*) from 0 μm to 0.1 μm, and almost remains unchanged in the gap (*w*) from 0.1 μm to 0.4 μm.

**Figure 4:**
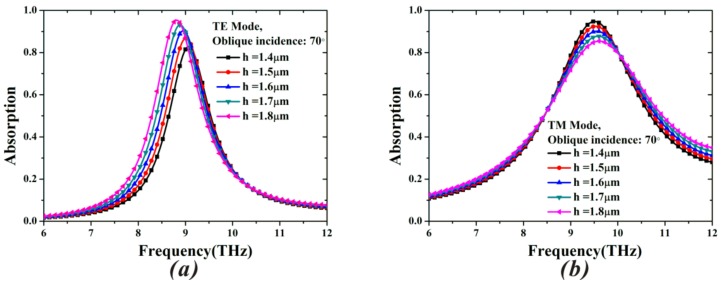
Absorption spectra of different dielectric thicknesses under an incident angle of 70° when the other parameters are fixed: (**a**) transverse electric (TE) mode and (**b**) transverse magnetic (TM) mode.

To determine the optimal thickness of the MA, we also investigate the impact of the different dielectric thicknesses on the properties of the resonance peak under oblique incidence of 70° for TE and TM mode in Figure 4a,b. For the TE case at 70° angle of incidence, when the dielectric thickness increases from 1.4 μm to 1.8 μm, the resonant peak shifts to the lower frequency. It agrees with the case at normal incidence in Figure 3d. However, the increased absorptivity disagrees with the cases in Figure 3d. By contrast, as shown in Figure 4b, the absorptance decreases when increasing the dielectric thicknesses in the case of TM mode, and the resonance frequency almost remains unchanged. Therefore, the absorptivity at 70° for both TE and TM mode can achieve greater than 90% only if the thickness (*h*) is 1.6 μm, and all other parameters are fixed. By the above analysis and discussion, a set of optimized geometric parameters is adopted in the simulation.

**Figure 5:**
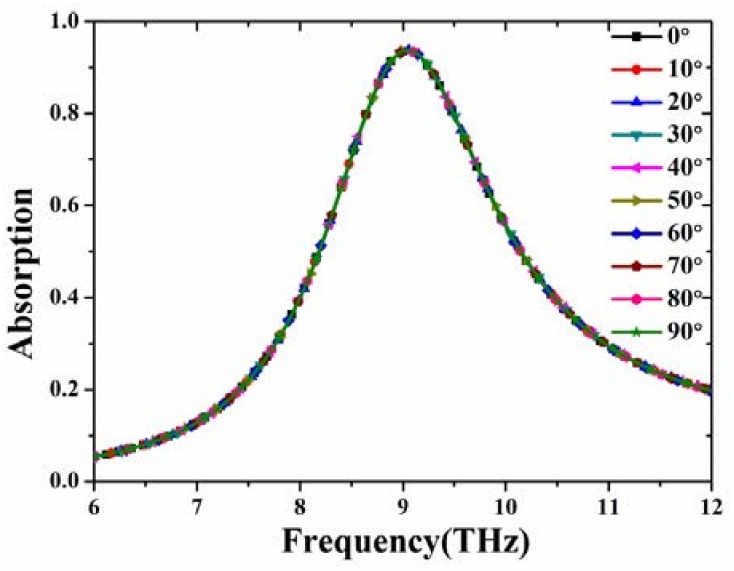
Simulated absorptivity under different polarization angles from 0° to 90° in steps of 10°.

**Figure 6:**
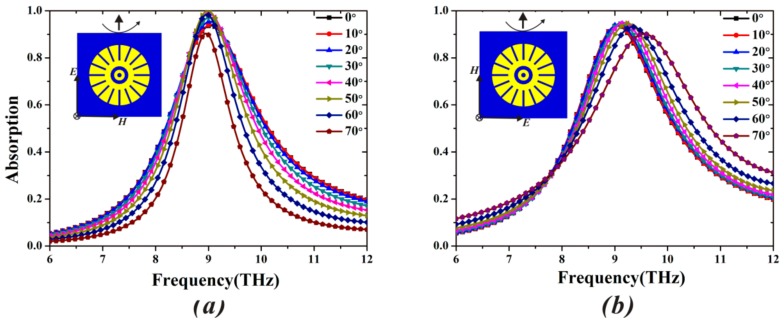
Simulated absorptivity under different incident angles from 0° to 70° for (**a**) TE mode and (**b**) TM mode.

**Figure 7:**
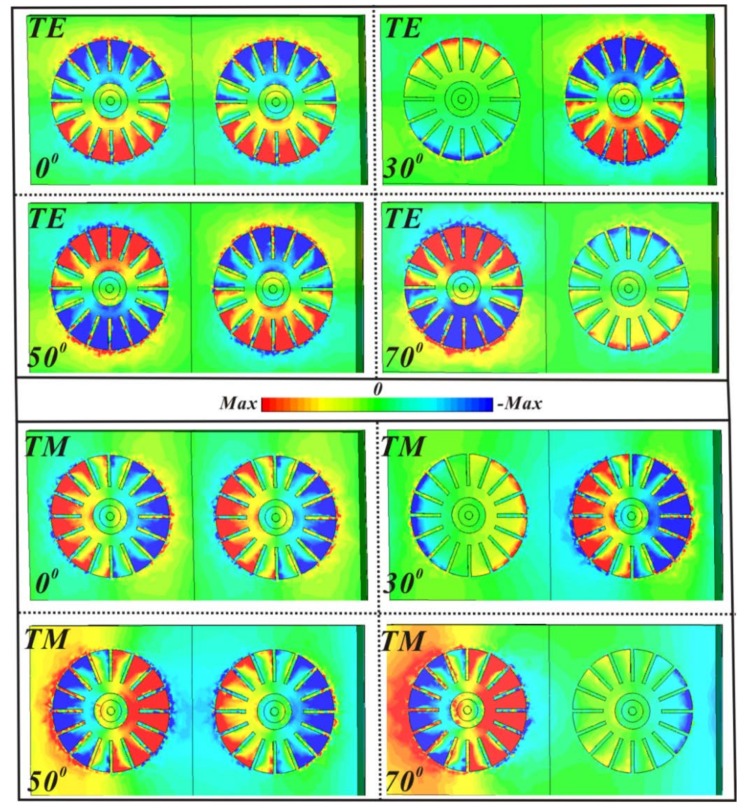
Distributions of Z-component of electric field (*E_z_*) at the top surface of sixteen-circular-sector resonator for TE and TM mode under the different incident angles of 0°, 30°, 50°, and 70°.

Based on the optimized sizes of the proposed MA, we study the absorption characteristics for various incident and polarization angles. In Figure 5, the absorptivity (93.7%) and resonance frequency (9.05 THz) of the proposed MA are all unchanged for various polarization angles ranging from 0° to 90° in steps of 10° [43]. This is due to the vertically and horizontally symmetrical structure of the MA. In Figure 6, the absorptivity achieves 90% for both TE and TM mode, even when the incident angle (*θ*) is up to 70°. It is observed from Figure 6a that the frequency variation in TE mode is only 0.25% when *θ* varies from 0° to 70°. Although the component of magnetic field gradually decreases with the increase of TE incident angle, and cannot efficiently induce antiparallel currents in the top and bottom layers, the electric dipole resonance remains strong in the different incident angles (see the TE case of Figure 7 below). Therefore, the MA can keep the high absorption for a wide range of incident angles. For the TM case, the frequency variation at 9.05 THz is 4.97% in all incident angles, as depicted in Figure 6b. By contrast, the shift of absorption peak under TM mode becomes bigger. In fact, at normal incidence, the electric dipole of each sixteen-circular-sector (SCR) resonator is triggered and in phase with each other (see the TM case of Figure 7 below). Thus, the positive and negative charges in the neighboring SCR resonators can attract each other, leading to formation of the attractive force. The attractive force along the $\vec{E}$ direction can weaken the restoring force inside the SCR resonator. Under oblique incidence, the electric dipole in each neighboring SCR resonators is out of phase, leading to the reduction of the attractive force (see the TM case of Figure 7 below) [44]. Therefore, the resonance frequency of the proposed MA experiences a blueshift in the case of TM mode.

In Table 1, we compare the performance of the proposed MA with that of the previous similar MAs for different incident angles. Many of these MAs have not exhibited polarization independence, although some MAs still maintain high absorption, even for the TM incident angle of 80°. For the TE case, the absorptivity of the MAs in the previous reports [22,28,29,33,34,40,45,46,47] is greater than 90% below oblique incidence of 60°. The proposed MA in the paper reaches more than 90% absorptivity, even when up to 70° for both TE and TM mode, and is not sensitive to the polarization angle.

To reveal the physical mechanism of the wide-angle absorption, the electric field (*E_z_*) distributions under different incident angles in the interface between the MA and air space are shown in Figure 7. Due to electromagnetic excitations, it is clearly observed that the fundamental dipole resonance is generated by the accumulation of the positive and negative charge, and the gap surface plasmons are also excited in the various incident angles of 0°, 30°, 50°, and 70° for TE and TM mode [48]. Moreover, for different incident angles, the intensity of accumulated charges is different, and the properties of the gap surface plasmons in each unit cell of MA vary accordingly. For the TE case, at normal incidence, the gap surface plasmons are observed, and the dipole circular-sector mode is anti-phase with the inner-ring mode and the gap surface plasmons [49,50]. At 30°, the intensity of the dipole circular-sector modes in the neighboring unit cell are inconsistent with each other, and the intensity in the right unit cell, which is as the same as that at normal incidence, is obviously higher than the case in the left unit cell. Additionally, the gap surface plasmons in the left unit cell are not excited. At 50°, the unit cell shows inverse electromagnetic properties with the neighboring one in the MA, and the electromagnetic intensity agrees with that at normal incidence. At 70°, the electromagnetic property of the left unit cell is stronger than that in the right unit cell, and its intensity is higher than that at normal incidence. In the left unit cell, the gap surface plasmons are also out phase with the dipole circular-sector mode and in phase with the inner-ring mode. Therefore, the proposed MA can maintain the high absorption of more than 90% even up to 70°. For the TM case, the properties of the dipole mode and gap surface plasmons are similar to those in TE mode. At 70°, the intensity of the gap surface plasmons between the neighboring unit cells is obviously different, leading to the shift of the absorption peak in Figure 6b.

**Figure 8:**
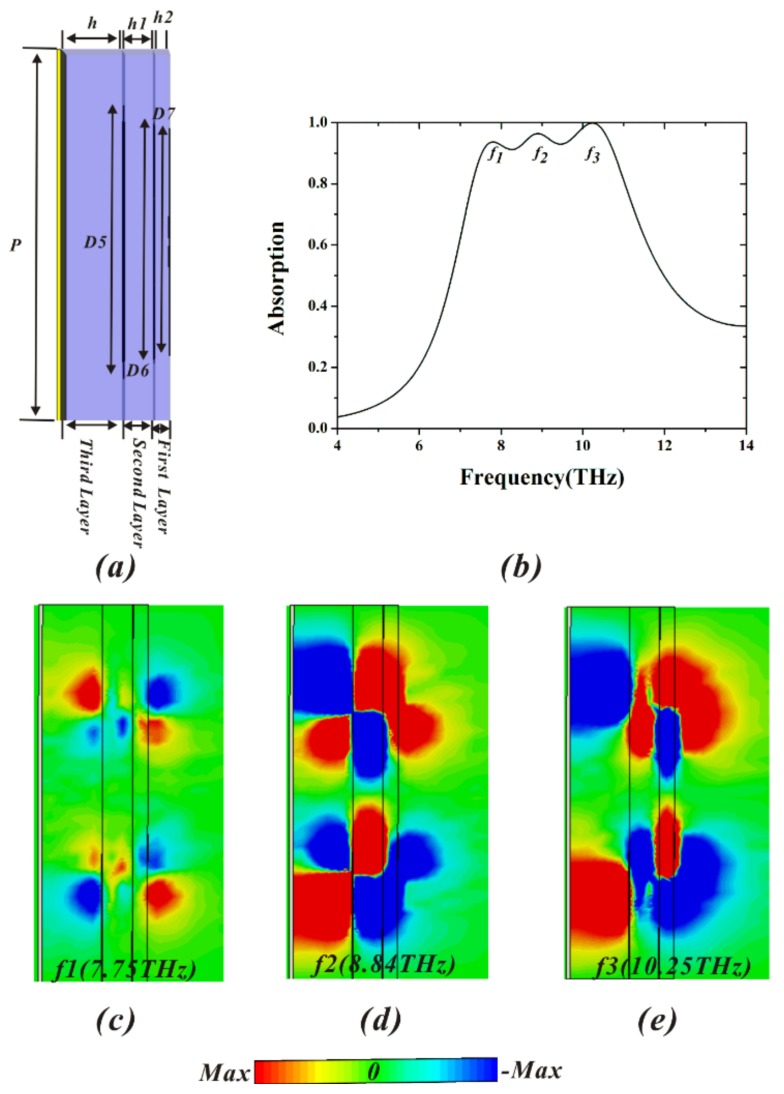
(**a**) Cross section of broadband MA, (**b**) absorptivity of broadband MA; distributions of y-component of magnetic field (*H_y_*) of three-layers structure for three resonance peaks on the cross section of y = 0: (**c**) 7.75 THz, (**d**) 8.84 THz, and (**e**) 10.25 THz.

We increase the bandwidth of the proposed MA by adopting multilayer structure in Figure 8. By tuning the size of resonator and the dielectric thickness, the perfect impedance matching between the multilayer structure and the free space at each neighboring resonant frequency is achieved, and further leads to the wideband absorption. In Figure 8a, the structure of the broadband MA consists of three layers and a metal ground. Each layer is composed of gold patterns and polymer substrate. *D5*, *D6*, and *D7* represent the outermost diameters of gold patterns in three layers from the bottom to the top, respectively. The final optimized geometric parameters are *D7* = 6 μm, *D6* = 6.4 μm, *D5* = 7.2 μm, *h2* = 0.4 μm, and *h1* = 0.8 μm, while the other parameters are identical to those in Figure 1a. The corresponding simulation result is shown in Figure 8b. Clearly, the designed MA achieves over 90% absorption in the deep infrared frequency range from 7.55 THz to 10.75 THz, and the absorption bandwidth is 3.2 THz. In addition, there are three strong absorption peaks at *f*_1_ = 7.75 THz (93.7%), *f*_2_ = 8.84 THz (96.4%), and *f*_3_ = 10.25 THz (99.9%). In particular, the absorptivity of the resonance frequency *f*_3_ can even reach the perfect absorption, with nearly 100%. To further understand the mechanism of its broadband absorption, the magnetic field (*H_y_*) distributions at the three resonance frequencies are shown in Figure 8c–e. It can be seen that the magnetic field of each layer strongly couple to each other, resulting in the hybridized mode [51]. Further, the hybridized mode contributes to three strong absorption peaks, leading to the broadband absorption.

## 4. Conclusions

We have demonstrated an angle- and polarization-insensitive MA using the sixteen-circular-sector resonator (SCR). At normal incidence, the absorptivity of the designed MA achieves 93.7% at 9.05 THz. The high absorption depends on both dielectric and ohmic losses. Then, the effects of different geometric parameters on the absorptivity are investigated in detail. Therefore, the resonant absorption can be adjusted by changing the geometric parameters of the unit cell. More importantly, the proposed MA can still keep more than 90% absorption for both TE and TM mode, even up to 70°. Especially for the TE case, the frequency variation is only 0.25% for large incident angles. The plasmon hybridizations are used to explain the mechanism of the wide-angle absorption. The distributions of the electric field (*E_z_*) reveal that the high absorption in a wide angular range originates from fundamental dipole and gap surface plasmon response of SCR structure. In addition, we also realize the broadband MA using multilayer structure in the deep infrared frequency range. Therefore, the designed MA can overcome the difficulty to achieve near-unity absorbance over a wide range of incident angles from 0° to 70° in both TE and TM mode. It is believed that the designed MA may have many promising potential applications in biosensors, imaging, and energy harvesting, especially demanding wide-angle incidence.

## Figures and Tables

**Table 1:** Performance comparison with THz MA of the previous reports.

Reference	Frequency Point/Band (THz)	Polarization Independence	Maximum of Incident Angle with the Absorptivity of More Than 90%	
			TE	TM
[22]	1.6	Not discussed	40°	80°
[28]	1.0	No	40°	40°
[29]	0.9	Not discussed	50°	50°
[33]	0.95	Yes	50°	50°
[34]	1.71	Yes	40°	60°
[40]	5	No	60°	80°
[45]	8.57	Not discussed	60°	60°
[46]	40	No	60°	80°
[47]	57.14	No	23°	50°
This study	9.05	Yes	70°	70°
